# Necrotizing neutrophilic dermatosis: A diagnostic challenge with a need for multi-disciplinary recognition, a case report

**DOI:** 10.1016/j.amsu.2020.07.037

**Published:** 2020-07-26

**Authors:** Asha Gowda, Luisa Christensen, Samantha Polly, Danny Barlev

**Affiliations:** Department of Dermatology, University Hospitals Cleveland Medical Center, Cleveland, OH, USA

**Keywords:** Sweet's syndrome, Necrotizing Sweet's syndrome, Necrotizing neutrophilic dermatosis, Necrotizing fasciitis, NND, Necrotizing neutrophilic dermatose, NF, Necrotizing fasciitis, SS, Sweet's syndrome

## Abstract

**Introduction:**

Necrotizing neutrophilic dermatoses can clinically resemble necrotizing fasciitis and therefore pose a diagnostic and therapeutic challenge. Given their similar presentations, misdiagnosis and inappropriate or delayed treatments are possible.

**Presentation of case:**

We discuss the case of a woman with acute myeloid leukemia who presented with fevers, chills, cough, and a leg wound. She underwent amputation of her lower extremity after she was presumed to have necrotizing fasciitis; however, symptoms persisted. She was ultimately diagnosed with and treated for necrotizing Sweet's syndrome with notable clinical improvement.

**Discussion:**

Both, necrotizing neutrophilic dermatoses and necrotizing fasciitis, grossly affect the skin and are associated with rapidly progressing systemic features including fevers, chills, leukocytosis, and elevated inflammatory markers. Recent literature in dermatology addresses these similarities and the appropriate approach to management; however, it is critical that medical and surgical subspecialties have an understanding of necrotizing neutrophilic dermatoses and their clinical presentations, diagnostic approaches, as well as therapeutic interventions. Familiarity with this entity can mitigate the risk of misdiagnosis, morbidity, and mortality.

**Conclusion:**

With this report, we seek to review the features that are suggestive of and aid in the diagnosis of necrotizing neutrophilic dermatoses to help prevent significant and avoidable morbidity.

## Highlights

• Necrotizing neutrophilic dermatoses are a rare skin condition that can easily be misdiagnosed as necrotizing fasciitis.
• Providers from specialties like internal medicine, oncology, and surgery may encounter necrotizing neutrophilic dermatosis.
• Familiarity with the management of necrotizing neutrophilic dermatoses can reduce avoidable morbidity.

## 1 Introduction

Neutrophilic dermatoses encompass a group of dermatologic conditions that have clinically distinct presentations with shared histopathologic features of dense sterile neutrophilic infiltration of the upper dermis [1]. The term necrotizing neutrophilic dermatoses (NND) was proposed to describe variants of neutrophilic dermatoses that resemble necrotizing fasciitis (NF) [2]. Given the resemblance of NND to NF, misdiagnosis and inappropriate or delayed treatment are possible, placing patients at risk for aggressive interventions and morbidity.

We describe a woman with NND, initially thought to have NF, who underwent unnecessary surgical intervention and a subsequent complicated hospital course. In addition to the case presented in this brief report, there are numerous cases described in the literature involving the misdiagnosis of NND and subsequent morbidity. Physicians outside of dermatology must be familiar with NND and distinguishing these from NF to prevent misdiagnoses and ensure appropriate medical treatment. Our case highlights the importance of recognizing NND and distinguishing them from necrotizing soft tissue infections. This work has been produced in line with the SCARE criteria [3].

## 2 Case report

A 66-year-old woman with hypothyroidism, Sjögren syndrome, essential thrombocytosis with transformation to proliferative myelofibrosis, and recent acute myeloid leukemia requiring chemotherapy with ruxolitinib, presented with fevers, chills, nausea, cough, and a bruise-like wound on her right leg that spontaneously developed three days prior. No other pertinent past medical, drug, or family history was noted. On arrival, she reported increasing erythema, enlargement, and new pain of the wound radiating to the calf. She denied trauma to the area, similar wounds in the past, or sensory changes. She was febrile to 39.5C with a blood pressure of 91/58 and heart rate of 86. Physical examination revealed induration, warmth, and erythema of the right leg sparing the digits, edema extending to the knee, and pain on palpation of the calf. Workup showed leukocytosis (53.9 × 10^9^/L), hemoglobin of 9.6 g/dL, thrombocytopenia (40 × 10^9^/L), mild transaminitis, lactate of 1.7 mmol/L, erythrocyte sedimentation rate of 61 mm/h, and C-reactive protein of 40.08 mg/dL. Screening for methicillin-resistant *Staphylococcus aureus* with nasal swab was negative. Chest radiograph showed perihilar infiltrates. Radiograph and ultrasound of the distal right leg were unremarkable without identifiable thromboses. Thus, broad-spectrum intravenous antibiotics were started for possible cellulitis.

**Fig. 1:**
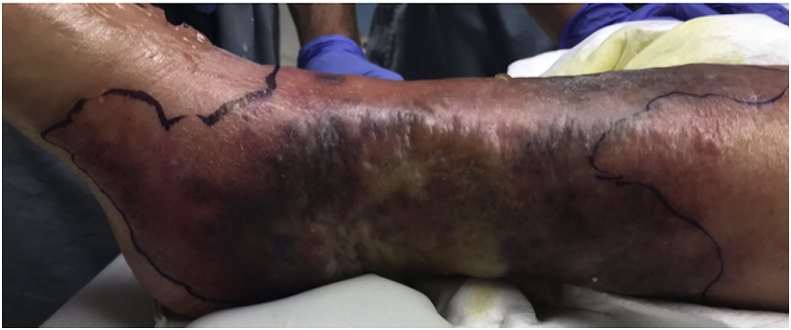
**Distal right lower extremity.** The distal right lower extremity with multiple ecchymotic and erythematous plaques with areas of serosanguinous drainage, without fluctuance or crepitus.

Over the next 48 hours, she remained febrile. She developed new-onset dyspnea requiring supplemental oxygen and multiple new ecchymotic, erythematous plaques on the right leg associated with intense pain and draining serosanguinous fluid without fluctuance or crepitus (Fig. 1). Blood and wound cultures from admission remained negative. Repeat imaging, including computed tomography and magnetic resonance imaging, showed diffuse soft tissue swelling around the calf concerning for cellulitis and/or lymphedema without evidence of osteomyelitis, deep intermyofascial edema concerning for fasciitis, and no signs of abscess. Hematology, infectious disease, podiatry, general surgery, orthopedic surgery, and vascular surgery teams were consulted. On day five, due to minimal improvement and acute concerns for necrotizing soft tissue infection and sepsis, she underwent below-the-knee amputation with clear margins for control of suspected infection.

Tissue specimens from the amputated leg obtained intraoperatively showed confluent sheets of neutrophils infiltrating the papillary and reticular dermis, with extension into the subcutaneous adipose tissue on histopathology. There was lobular and predominantly septal involvement, as well as fascial involvement. Additionally, there was marked papillary dermal edema, areas of ulceration, and hemorrhage, without intravascular thrombi. Gram stain for organisms and CD34 immunostaining for atypical mononuclear infiltrate were negative, findings suggestive of possible infection, bullous Sweet's syndrome (SS), or pyoderma gangrenosum.

Despite amputation and use of broad-spectrum antibiotics, her clinical picture worsened. She continued to have dyspnea with a nonproductive cough, progression of erythema at the site of amputation, anemia requiring blood transfusions, and worsening bilateral lung opacities and aeration on imaging. Workup for anti-phospholipid syndrome was negative. Blood, wound, and tissue cultures also remained negative for bacterial, viral, fungal, and acid-fast organisms. Therefore, she had repeat intervention and revision of the amputation. Intraoperative tissue biopsy from the stump revealed histopathology similar to that observed in the initial amputated specimen. At this time, dermatology also was consulted for further workup.

On day thirteen, she developed a new tender, red plaque on the left forearm. Clinically and histopathologically more consistent with a neutrophilic dermatoses. Given her clinicopathologic findings, she was ultimately diagnosed with necrotizing SS. Treatment with oral prednisone (1 mg/kg) led to rapid improvement of the new lesion. Her respiratory status also began to improve, and within six days of initiating steroids, she no longer required supplemental oxygen. Given her hematologic malignancy, immunocompromised state, and anemia, she was discharged with a slow taper of steroids followed by transition to intravenous immunoglobulin.

## 3 Discussion

To diagnose SS, or acute febrile neutrophilic dermatosis, the major criteria and two of the four minor criteria must be met (Table 1) [4,5]. Typically, patients present with erythematous plaques or nodules favoring the head, neck, and distal extremities, which on histopathology, reveal a dense neutrophilic infiltrate involving the dermis and occasionally, subcutaneous fat [2]. Patients also commonly present with fever and leukocytosis, and have dramatic improvement with high-dose prednisone.

**Table 1:** Diagnostic criteria for Sweet's syndrome [5].

Major criteria
1. Sudden onset of tender, erythematous plaques or nodules 2. Dense neutrophilic infiltrate in the upper dermis without evidence of leukocytoclastic vasculitis
**Minor criteria**
1. Fever (38C or higher), arthralgia, or malaise 2. Rapid response to systemic corticosteroids 3. Abnormal laboratory values at presentation (three or more of the following: leukocyte count > 8000; greater than 70% neutrophils; elevated C-reactive protein; erythrocyte sedimentation rate > 20mm/h) 4. Association with underlying malignancy, inflammatory disease, pregnancy; or, preceding infection or vaccination

NND, such as necrotizing SS [2], present with significant progression of cutaneous disease with systemic symptoms, which can resemble NF clinically and histopathologically [6]. As opposed to typical SS, in which neutrophilic infiltration primarily involves the dermis and occasionally the subcutaneous fat, necrotizing SS is a deeper process involving the fascia and skeletal musculature [7].

Approximately 21% of SS cases are malignancy-related, particularly hematological malignancies [8,9]. Within this subcategory, acute myeloid leukemia is the most commonly-reported association, followed by myelodysplastic syndrome [[8], [9], [10]]. Interestingly, atypical presentations of SS occur more frequently in patients with myeloproliferative disorders [11,12]. Unlike classic SS, malignancy-associated SS can present with bullae or pustules that can ulcerate and may resemble fungal infections or necrotizing soft tissue diseases like NF [9,13]. One theory suggests that tumor antigens from myeloproliferative disorders set off a reactive process leading to amplification of pro-inflammatory cytokines, such as interleukin-1 [9]. These signaling molecules are typically found in neutrophilic dermatoses and ultimately lead to an aggressive presentation with extracutaneous involvement such as renal, hepatic, or cardiac dysfunction, with possible progression to multi-organ system failure and death [9,13,14].

Our patient's presentation was concerning for systemic inflammatory response syndrome, thought to be secondary to a skin infection. Given the concerns for NF, she received prompt treatment with systemic antibiotics and surgical intervention with tissue debridement and limb amputation as life-saving measures. Unfortunately, as observed in this case, the cutaneous and systemic features of NF can mimic non-infectious dermatologic conditions with multisystem involvement, leading to delayed diagnosis. Sanchez et al. proposed a set of diagnostic criteria to assist with the differentiation of NND from NF, which are included in Table 2 along with features noted by other clinicians [6,15].

**Table 2:** Features supportive of necrotizing neutrophilic dermatoses [6,10,15].

Clinical features
Presence of erythematous or ulcerative plaques with violaceous borders Multisystem involvement resembling shock with fevers and lack of an identifiable infectious source Lack of crepitus
**Histopathologic features**
Neutrophil infiltrations and necrosis involving the fascia and/or muscle
**Treatment course**
A lack of clinical improvement or worsening in clinical picture seen with antibiotics or surgical intervention Rapid response to systemic steroids
**Presence of other known comorbidities or associations**
Malignancies Hematologic disorders Pregnancy Inflammatory diseases (Inflammatory bowel disease) Medications (Granulocyte-colony stimulating factor, Radiation therapy) History of pathergy, or development of or worsening of skin disease in areas of minor trauma (eg. sites of debridement or injections)

Although our patient's presentation was suggestive of NF with systemic involvement, her blood and wound cultures yielded no growth of infectious organisms, and she ultimately was diagnosed with necrotizing SS. Risk factors included our patient's history of acute myeloid leukemia and prior treatment with ruxolitinib, which has been reported to be associated with SS in patients with myelofibrosis [16,17]. With the lungs commonly being affected in systemic SS [13], her symptoms of cough and dyspnea, which did not improve with antibiotics and improved with systemic steroids, also support the diagnosis of necrotizing SS. Local progression of her cutaneous findings after surgical debridement was likely a manifestation of pathergy, the development of new or exacerbation of existing lesions secondary to trauma, which is classically seen with neutrophilic dermatoses.

Several cases of NND misdiagnosed as NF have been reported in the dermatologic literature [2,6,18,19]. A case series involving 54 patients with NND revealed that 94% were initially given a diagnosis of NF [6]. This alarming rate of misdiagnosis underscores the pressing need to educate clinicians about NND. More worrisome than the misdiagnosis itself is the subsequent mismanagement that it may lead to. In the same case series by Sanchez et al., 77.8% of patients with NND underwent unnecessary surgical debridement and amputations [6].

NND are rare and thus not usually considered by other medical specialties. Given that the associated risk factors for neutrophilic dermatoses include malignancy, hematologic disorders, and inflammatory bowel disease, medical specialists, such as oncologists, hematologists, and gastroenterologists, who may care for the patients likely to develop NND should be prepared to recognize its presenting features. Similarly, considering the overlapping features of NND and NF, infectious disease and surgical specialties should also be well-acquainted with NND, the clinical characteristics distinguishing it from NF, and its treatment options. Prompt recognition of this entity with initiation of systemic therapy can prevent unnecessary surgical interventions and morbidity, and so we hope to illustrate with our case, the importance of early recognition of NND by other medical specialties. Additionally, we encourage having a low threshold for dermatology consultation for patients presenting with soft-tissue necrosis without evidence of an infectious etiology.

## 4 Conclusion

Necrotizing neutrophilic dermatoses can be a diagnostic challenge, especially given its resemblance to NF, which has a drastically distinct and more emergent approach to treatment. As seen in this report, NND may be encountered in the clinical setting by a variety of healthcare providers; thus, it is crucial for medical and surgical specialties to be familiar with NND and include this diagnosis in the differential for clinically-similar diseases, like necrotizing fasciitis, to prevent avoidable morbidity.

## Ethical approval

This study was submitted to the institutional review board (IRB). The IRB determined that this study did not require IRB review and/or approval for its completion.

## Sources of funding

This study has received no funding.

## Author contribution

Study concept or design – AG, LC, SP.

Data collection – AG, LC, SP, DB.

Data interpretation – AG, LC, SP, DB.

Literature review – AG, LC, SP.

Preparation of photographs and paper – AG, LC, SP.

Editing of the paper – AG, LC, SP, DB.

## Trial registry number

This is a case report which does not require a registry.

## Guarantor

Drs. Asha Gowda, Luisa Christensen, Samantha Polly, Danny Barlev.

## Consent

The patient described in this case report passed away prior to the time period during which this case report was decided to be written. Thus, on several occasions, the family member recorded was contacted to request and obtain consent as the patient's next of kin on the patient's behalf, however without success. Subsequently, ethical approval by the institutional review board (IRB) was requested for this report. The IRB determined that this study did not require IRB review and/or approval for its completion Supplemental information has been provided to outline details of the written patient consent required for publication. Given the circumstances, write-up of this case without formal consent was permitted by the IRB.

This study does not include any patient identifying information.

## Patient consent

The patient described in this case report passed away prior to the time period during which this case report was decided to be written. Thus, on several occasions, the family member recorded was contacted to request and obtain consent as the patient's next of kin on the patient's behalf, however without success. Subsequently, ethical approval by the institutional review board (IRB) was requested for this report. The IRB determined that this study did not require IRB review and/or approval for its completion Supplemental information has been provided to outline details of the written patient consent required for publication. Given the circumstances, write-up of this case without formal consent was permitted by the IRB.

This study does not include any patient identifying information.

## Provenance and peer review

Not commissioned, externally peer reviewed.

## Authors’ disclaimer

The authors indicate no potential conflicts of interest. No funding was required for this report. This manuscript has not been previously published and is not being considered for publication elsewhere.

## Declaration of competing interest

The authors have no potential conflicts of interest to disclose.
